# Does metabolite channeling accelerate enzyme-catalyzed cascade reactions?

**DOI:** 10.1371/journal.pone.0172673

**Published:** 2017-02-24

**Authors:** Liubov Poshyvailo, Eric von Lieres, Svyatoslav Kondrat

**Affiliations:** 1 Forschungszentrum Jülich, IBG-1: Biotechnology, Jülich, Germany; 2 Forschungszentrum Jülich, IEK-7: Institute of Energy and Climate Research, Jülich, Germany; Virginia Polytechnic Institute and State University, UNITED STATES

## Abstract

Metabolite or substrate channeling is a direct transfer of metabolites from one enzyme to the next enzyme in a cascade. Among many potential advantages of substrate channeling, acceleration of the total reaction rate is considered as one of the most important and self-evident. However, using a simple model, supported by stochastic simulations, we show that it is not always the case; particularly at long times (*i.e.* in steady state) and high substrate concentrations, a channeled reaction cannot be faster, and can even be slower, than the original non-channeled cascade reaction. In addition we show that increasing the degree of channeling may lead to an increase of the metabolite pool size. We substantiate that the main advantage of channeling likely lies in protecting metabolites from degradation or competing side reactions.

## Introduction

Enzyme-catalyzed cascade reactions play a crucial role in all physical-chemical processes in living systems and have numerous biotechnological applications. For instance, in glycolysis, which is probably the most ubiquitously present metabolic pathway in living organisms, glucose is transformed into pyruvate by ten sequential enzyme-catalyzed reactions. Enzymatic cascades are widely used in pharmaceutical and chemical industry, in medical research and other fields.

Enzymes are proteins with specific catalytic activities. As other catalysts, they do not change the chemical equilibrium, but increase the rate with which the equilibrium is achieved. Unlike most catalysts, enzymes are typically very specific and often accept only one substrate type for catalysis. Many enzyme-catalyzed reactions follow the Michaelis-Menten kinetics [1, 2], in which an enzyme and a substrate first form a complex, and then the substrate is converted into the product and released into the bulk solution. Other mechanisms are also known [3–7].

Metabolite or substrate channeling means a direct transfer of the product of one enzyme to another in a cascade reaction, without releasing it into the bulk solution [8–12]. This is realized through formation of enzyme-enzyme complexes, also called enzyme assemblies or metabolons. In these complexes the reaction intermediates are transfered from the active site of one enzyme to the active site of another enzyme either inside a physical tunnel [11–14] or along an ‘electrostatic highway’ [11, 12, 15, 16] (both called direct channeling), or diffusionally, but along a shortened path, in which case it is termed ‘channeling by proximity’ [17–22].

Although strong experimental evidence now exists in support of enzyme-enzyme complexes *in vivo* [23–27], the role of channeling, its presence and physiological significance in living cells have long been an abiding subject of debates [28–31]. In biotechnology, on the other hand, channeling is considered as an important method to reduce the amount of intermediate substances in solutions (*e.g.* to protect cells or bioreactors from toxic intermediates) and to accelerate the overall reaction rates [20–22, 32–34]. Indeed, it appears to be a widely accepted paradigm in biotechnology that channeling of intermediates accelerates cascade reactions. Thus, Zhang writes [32], when listing the benefits of channeling: *“In addition to accelerating reaction rates through substrate channeling, potential benefits of complexes include…”*, *i.e.* the reaction acceleration is treated as self-evident and is not even discussed. But there are also other views. For instance, Wheeldon *et al*. say in a very recent review article that [8] *“the overall rate of a cascade reaction is not a function of channeling.”* Here, we attempt to clarify whether substrate channeling influences the velocity of enzyme-catalyzed cascade reactions, and if so how and when.

For simplicity, we consider a cascade consisting of just two enzyme-catalyzed reactions,

$$\text{S}+{\text{E}}_{1}\overset{a_{1}}{\underset{d_{1}}{⇌}}{\text{SE}}_{\text{1}}\overset{k_{1}}{\to }{\text{E}}_{1}+\text{I},$$ (1a)

$$\text{I}+{\text{E}}_{2}\overset{a_{2}}{\underset{d_{2}}{⇌}}{\text{IE}}_{2}\overset{k_{2}}{\to }{\text{E}}_{2}+\text{P},$$ (1b)

in which the product of the first reaction (intermediate, I) is the substrate for the second. Here *a*_*i*_ and *d*_*i*_ are *macroscopic* association and dissociation rate constants, respectively, *k*_*i*_ is the turnover number of *i*’th enzyme, and we have assumed the standard Michaelis-Menten kinetics. In defiance of its simplicity, the Michaelis-Menten model describes well many enzyme-catalyzed reactions [7], including some reactions requiring co-factors, provided the co-factors are in abundance (see Supporting Information and the Discussion below).

We suppose now that in cascade reaction Eq (1) the intermediates are channeled between enzymes E_1_ and E_2_, through the formation of a complex E_12_ = E_1_E_2_. A particular mechanism of channeling is not important for our purposes, and will not be discussed here, instead we consider the following simple model for channeling

$$\text{S}+{\text{E}}_{12}\overset{d_{ch}}{\underset{a_{ch}}{⇌}}{\text{SE}}_{12}\overset{k_{1}^{ch}}{\to }{\text{IE}}_{12}\overset{k_{ch}}{\to }{\text{E}}_{12}\text{I}\overset{k_{2}^{ch}}{\to }{\text{E}}_{12}+\text{P},$$ (2)

where SE_12_ (IE_12_) denotes a complex in which S (I) is attached to the active site of E_1_, while E_12_ I is a complex in which an intermediate is at the active site of E_2_; *k*_*ch*_ is a channeling rate constant, *i.e.* it is inverse of the time needed by an intermediate to travel from the active site of E_1_ to the active site of E_2_ in the E_12_ complex. For instance, Brownian dynamics simulations predict for the total journey time of a 4-hydroxy-2-ketovalerate in the intermolecular channel of a bifunctional DmpFG enzyme $k_{ch}^{-1}\approx 25\text{ns}$ [13].

We assume for simplicity that the enzyme-catalyzed reactions and the channeling process are both irreversible, and that the enzyme complex can process one substrate molecule at a time. Extensions are possible and will be discussed below.

In what follows we assume that all non-diffusional (microscopic or intrinsic) rate constants are not altered when the enzymes form a complex. While this assumption might not generally be true, we note that if the overall reaction velocity changes because one of the microscopic rate constants differs in a complex and for the free enzymes, it is then this rate constant that is the cause of the change, rather than channeling *per se*. In this work we are solely interested in the effect of channeling as such, and for this reason we assume that $k_{i}=k_{i}^{ch}$ in Eqs (1) and (2). Likewise, we will assume that microscopic (intrinsic) contributions to the macroscopic association/dissociation rate constants (*a*_1_, *d*_1_, and *a*_*ch*_, *d*_*ch*_) are the same for the channeled and non-channeled reactions (see below).

It is possible that enzymes are more stable in an enzyme-enzyme complex than alone. While this can undoubtedly be an important benefit of complex formation, it says little about the advantage of channeling as such. Indeed, in this case such a stabilization can potentially be achieved by complexing each enzyme separately with non-active proteins, through immobilization [35], or by placing enzymes in nanocages [36].

Channeling model (2) may or may not accurately describe the kinetics of real enzyme complexes. In particular, this is not a valid model for the majority of proximity channeling techniques (we shall consider this case in a separate article). However, this is a simple, analytically tractable model that will help us develop new physical insights and reach conclusions in fact more general than the model itself offers. To compare the overall reaction rates within this model with the rates of the original non-channeled cascade reaction, Eq (1), we assume that both systems are well stirred and formulate a set of ordinary differential equations based on the Michaelis-Menten kinetics. We support these results by Brownian dynamics simulations by which we determine the rates of diffusion-limited channeled and non-channeled reactions.

## Results

There are three typical situations occurring *in vivo* and in biocatalytic experiments. For cellular systems, it is reasonable to assume that the concentration of substrate molecules in cells remains (roughly) constant under pleasurable conditions. In biocatalytic laboratory experiments, a popular setup is a batch reactor, in which substrate molecules are well mixed with enzymes, and the reactions are observed without any further addition of substrates. In semi-batch or continuous stirred-tank or flow reactors, the substrate molecules are continuously supplied to the system, often with a constant rate.

In the latter case it is clear that when the substrate supply velocity is not too high (that is, it does not exceed the maximum rate set by the enzyme capability, *cf.* Eq (7)), then the mass conservation implies that the rate of product formation at long times, *i.e.* in steady state, is equal to the rate of substrate supply. Obviously this is so for both channeled and non-channeled systems. Thus, we shall not discuss this case here. Instead, we focus on the case when the substrate concentration is kept constant in the system (cellular conditions), and comment on the batch reactors later.

We consider first long-time steady-state behaviour. In this case, it is straightforward to find for the product formation velocity of the channeled system (see Methods)

$$\begin{array}{c} v_{ch}=\frac{k_{1}k_{ch}k_{2}\left[S\right]\left[E_{12}\right]}{k_{ch}\left\{K_{M}^{ch}k_{2}+\left[S\right]\left(k_{1}+k_{2}\right)\right\}+\left[S\right]k_{1}k_{2}}, \end{array}$$ (3)

where $K_{M}^{ch}=\left(k_{1}+d_{ch}\right)/a_{ch}$ is the Michaelis-Menten constant. Here [E_12_] is the *total* concentration of the enzyme complexes, and, likewise, [E_*i*_] below is the total concentration of enzyme *i*.

The product formation velocity of the non-channeled system depends on the relation between the rates of individual reactions and the enzyme and substrate concentrations. When the first reaction in Eq (1) is rate-limiting, then the concentration of intermediates is constant at long times, which means that *d*[I]/*dt* = 0, and hence we have

$$\begin{array}{c} v_{non}=\frac{k_{1}\left[S\right]\left[E_{1}\right]}{K_{M}^{\left(1\right)}+\left[S\right]}, \end{array}$$ (4)

where $K_{M}^{\left(1\right)}=\left(k_{1}+d_{1}\right)/a_{1}$ is the Michaelis-Menten constant of the first reaction. In the opposite case, when the second reaction is rate-limiting, the first reaction at long times will produce an excess amount of intermediates, and the concentration of intermediates will increase with time. This means that [I] → ∞ as *t* → ∞, and we obtain

$$\begin{array}{c} v_{non}=k_{2}\left[E_{2}\right]\left[S\right]. \end{array}$$ (5)

The borderline between these two regimes is at the substrate concentration

$$\begin{array}{c} {\left[S\right]}_{\text{threshold}}=\frac{K_{M}^{\left(1\right)}k_{2}\left[E_{2}\right]}{k_{1}\left[E_{1}\right]-k_{2}\left[E_{2}\right]}. \end{array}$$ (6)

For concentrations above [S]_threshold_, the first reaction outperforms the second, and the amount of intermediates will increase with time; in this case the rate of product formation is given by Eq (5). For [S] < [S]_threshold_, the second reaction is fast enough to convert the available intermediates, the concentration of intermediates is therefore always finite, and the production rate is given by Eq (4).

We focus now on the case when the first reaction is rate-limiting, and consider first two limiting cases of high and low substrate concentrations. For high substrate concentrations ($\left[S\right]\gg K_{M}^{\left(1\right)}$ and $\left[S\right]\gg K_{M}^{ch}/(1+k_{1}/k_{2}+k_{1}/k_{ch})$ but below [S]_threshold_) we get

$$\begin{array}{c} v_{non}^{\left(max\right)}=k_{1}\left[E_{1}\right] \end{array}$$ (7a)

and

$$\begin{array}{c} v_{ch}^{\left(max\right)}={\left(\frac{1}{k_{1}}+\frac{1}{k_{ch}}+\frac{1}{k_{2}}\right)}^{-1}\left[E_{12}\right]. \end{array}$$ (7b)

This equation means that metabolite channeling *slows down* the overall reaction, provided of course that [E_1_] = [E_12_], which we assume is true for a fair comparison. Note that $v_{\alpha }^{\left(max\right)}$ (where *α* = {*ch*,*non*}) are the maximum achievable rates determined by the enzyme’s capabilities. (Incidentally, in the case that the second reaction in a cascade is rate-limiting we have $v_{non}^{\left(max\right)}=k_{2}\left[E_{2}\right]<v_{ch}^{\left(max\right)}$ for [E_2_] = [E_12_].)

At low substrate concentrations we have

$$\begin{array}{c} v_{non}=\frac{k_{1}\left[E_{1}\right]\left[S\right]}{K_{M}^{\left(1\right)}}\;\text{and}\;v_{ch}=\frac{k_{1}\left[E_{12}\right]\left[S\right]}{K_{M}^{ch}}, \end{array}$$ (8)

and hence whether the overall reaction is accelerated or decelerated by channeling is determined by the ratio $K_{M}^{ch}/K_{M}^{\left(1\right)}$.

### Extent of diffusion-control

To study the case of low and intermediate substrate concentrations, we first split all association rate constants into their diffusional and microscopic (intrinsic) parts [37–39]; that is, we present an association constant *a* as the sum

$$\begin{array}{c} a^{-1}=k_{a}^{-1}+k_{D}^{-1}, \end{array}$$ (9)

and the corresponding dissociation constant as [38] *d* = *k*_*d*_/(1 + *k*_*a*_/*k*_*D*_). Here *k*_*a*_ and *k*_*d*_ are *microscopic* (intrinsic) rate constants and *k*_*D*_ ≈ 4*πDR* the rate due to diffusion [40], where *D* is the mutual diffusion coefficient and *R* the reaction radius. Then, following Fange *et al*. [41], we introduce the degree of diffusion control

$$\begin{array}{c} \gamma =k_{a}/k_{D}. \end{array}$$ (10)

If *γ* ≫ 1, then *k*_*a*_ ≫ *k*_*D*_ and the reaction is diffusion limited (*a* ≈ *k*_*D*_). In the opposite case, *γ* ≪ 1, we have *a* ≈ *k*_*a*_ and the reaction is limited by the intrinsic (microscopic) rate.

We now assume that, in addition to the enzyme’s turnover numbers $k_{i}^{ch}=k_{i}$, also the *microscopic* association/dissociation rate constants of the channeled and non-channeled reactions are equal (*i.e.* $k_{a}^{ch}=k_{a}^{\left(1\right)}$ and $k_{d}^{ch}=k_{d}^{\left(1\right)}$), and investigate how channeling affects the production velocity, depending on the degree of diffusion control of these reactions.

**Fig 1:**
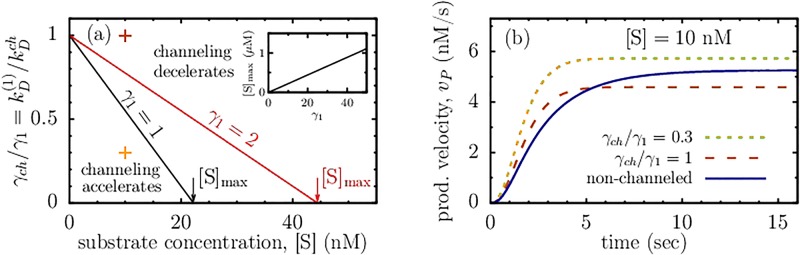
Effect of channeling in systems with constant substrate concentrations. (a) Channeling diagram showing the regions in which channeling accelerates/decelerates enzyme-catalyzed tandem reactions in steady state. These regions are separated by lines calculated for different degrees of diffusion control of the first reaction in the tandem, *γ*_1_. The degree of diffusion control of the enzyme complex is denoted by *γ*_*ch*_. In general, the degree of diffusion control is *γ* = *k*_*a*_/*k*_*D*_, where *k*_*a*_ is the intrinsic (microscopic) association rate constant and *k*_*D*_ is the rate due to diffusion. Then the *microscopic* dissociation rate constant is *k*_*d*_ = *d*(1 + *γ*), where *d* is the total dissociation constant; similarly, the *microscopic* association constant is *k*_*a*_ = *a*(1 + *γ*), where *a* is the total association constant. *γ*, *a* and *d* can all be different for channeled and non-channeled reactions (*i.e.* *a*_1_ ≠ *a*_*ch*_ *etc.*), but we have assumed that the microscopic rate constants are equal, *i.e.* $k_{a}^{ch}=k_{a}^{\left(1\right)}$ and $k_{d}^{ch}=k_{d}^{\left(1\right)}$. [S]_max_ denotes the substrate concentration above which channeling decelerates the tandem reaction independently of the value of *γ*_*ch*_. The inset shows how [S]_max_ depends on *γ*_1_. (b) Product formation velocity, *v*_*P*_, for the non-channeled reaction and for two channeled reactions with different *γ*_*ch*_/*γ*_1_, shown by symbols in (a). In all plots, the intrinsic association and dissociation rate constants are $k_{a}^{\left(i\right)}=k_{a}^{ch}=0.027{\text{ nM}}^{-1}{\text{ s}}^{-1}$ and $k_{d}^{\left(i\right)}=k_{d}^{ch}=1.35{\text{ s}}^{-1}$, where *i* = 1,2. The enzyme’s turnover numbers are $k_{i}=k_{i}^{ch}=1.5{\text{ s}}^{-1}$. These rate constants correspond to the first two reactions in the MAPK pathway [42]. The rate of channeling is *k*_*ch*_ = 1 s^−1^ (see Eq (2)).

A diagram in Fig 1a shows the regions in which channeling accelerates/decelerates tandem reactions in steady-state. In accord with Eq (7), the non-channeled reaction is faster at high substrate concentrations, [S] > [S]_max_, at which the concentration of *intermediates* is sufficiently high to ensure its fast rate. At low [S], the channeled reaction can only be faster if channeling reduces the degree of diffusion control, that is if *γ*_*ch*_ < *γ*_1_ and hence $k_{D}^{ch}>k_{D}^{\left(1\right)}$. This is clear as in the steady state the rates are essentially determined by the first reaction in a cascade (in the case discussed, *i.e.* when the first reaction is rate limiting), and the only way to accelerate the overall rate is to increase the rate due to diffusion (for otherwise equal parameters). The region in which channeling provides higher rates increases as *γ*_1_ increases. Hence the maximum substrate concentration, [S]_max_, below which the metabolite channeling has the potential to accelerate the reaction, also increases with *γ*_1_ (the inset in Fig 1a).

Fig 1b shows the production velocity, *v*_*P*_, as a function of time for the channeled and non-channeled systems. In accord with the numerous experimental studies [20–22, 43], in all cases we looked at, *v*_*P*_ was found higher for the channeled system at short times. The reason is that initially the concentration of intermediates is low, and hence the non-channeled system needs more time to advance to its steady state. In the long run, however, the overall production rate of a system with channeling can be lower for sufficiently large *γ*_*ch*_.

### Batch reactor

In a batch reactor, substrate molecules and enzymes are mixed and the reaction is observed without any further addition of substrate molecules. In other words, the substrate supply velocity is zero. To compare the systems with and without channeling in this case, we look at the time needed by each system to reach the product concentration [P] = *α*[S]_0_, where [S]_0_ is the initial substrate concentration and *α* a comparison parameter.

**Fig 2:**
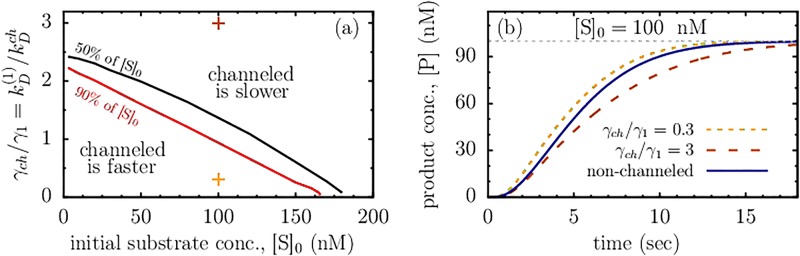
Effect of channeling in batch reactors. (a) Channeling diagram showing the regions in which the channeled reaction transforms the shown amount of the substrate (*α*[S]_0_) faster or slower than the non-channeled tandem reaction. The demarcation lines of this diagram were calculated by comparing the times needed by the channeled and non-channeled systems to achieve the product concentration [P] = *α*[S]_0_; these times are equal on the demarcation lines. The values of *α* expressed as percentage of [S]_0_ are denoted on the figure. *γ*_1_ and *γ*_*ch*_ are the degrees of diffusion control of the first reaction in the cascade and of the channeled reaction, respectively (see Eq (10) and Fig 1). (b) Product concentration as a function of time for the non-channeled reaction and for the channeled reactions with different *γ*_*ch*_/*γ*_1_, shown by symbols in (a). The thin vertical line denotes [P](*t* = ∞) = [S]_0_. We have used *γ*_1_ = *γ*_2_ = 1 for the first and second reactions in the cascade to produce all plots. The remaining parameters are the same as in Fig 1.

The results are summarized in Fig 2a and typical time-dependent concentration profiles are shown in Fig 2b. The diagram in Fig 2a is drawn in the plane of the *initial* substrate concentration ([S]_0_) and the degree of diffusion control of the channeled reaction, and has a topology similar to Fig 1a. It shows that the region in which the channeled reaction transforms substrates faster is narrow and essentially bound to low values of [S]_0_. This region shrinks as we increase the comparison parameter *α*. This emphasizes that we compare the systems that are not in steady states, thus the diagram also reflects how these systems would approach the steady states, were the substrate supplied to them.

### Diffusion-limited rates

**Fig 3:**
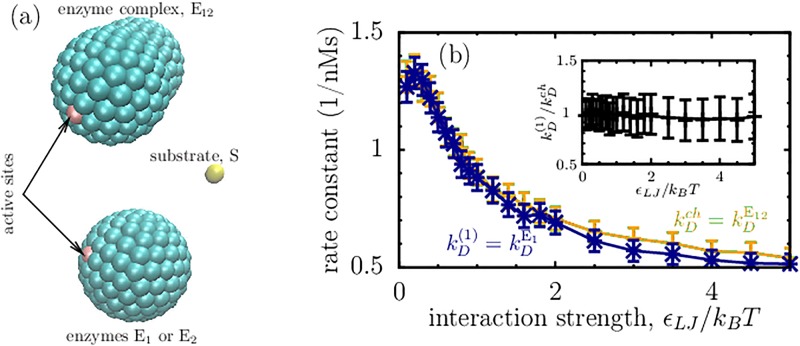
Single enzymes *versus* enzyme-enzyme complexes. (a) Enzymes E_1_ and E_2_ consist of Lennard-Jones (LJ) spheres of radius ≈0.37 nm placed on the surface of a sphere of radius 1.5 nm, where one of the LJ-spheres is treated as an active site. An enzyme complex (E_12_ = E_1_E_2_) is constructed by merging E_1_ and E_2_ together such that the active site of E_1_ is located on the axis connecting the centers of E_1_ and E_2_. The center-to-center distance between E_1_ and E_2_ is 1.4 nm. The substrate for E_1_ and E_12_ is a LJ-sphere of radius 0.4 nm. (b) Rates of the diffusion-limited enzyme-catalyzed reactions for the first enzyme in a tandem reaction (denoted by $k_{D}^{E_{1}}\equiv k_{D}^{\left(1\right)}$) and for the enzyme-enzyme complex (denoted by $k_{D}^{E_{12}}\equiv k_{D}^{ch}$). The rates are shown as functions of the strength of the LJ potential between the substrate molecule and the LJ particles of the enzymes (*ϵ*_*LJ*_). These rates were calculated by using Brownian dynamics simulations (see Methods).

Figs 1 and 2 show that channeling can accelerate or decelerate cascade reactions depending on how the degree of diffusion control (or the reaction rate due to diffusion) changes when the enzymes form a complex. To gain some insight into this dependence, we compare the diffusion-limited association rates for a single enzyme and for an enzyme-enzyme complex, *i.e.* $k_{D}^{ch}$ and $k_{D}^{\left(1\right)}$ in Eq (9). To model this situation, we consider a complex obtained by merging two identical spherical enzymes, as shown in Fig 3a. Each enzyme consists of Lennard-Jones (LJ) particles placed on the enzyme surface, while the substrate is modelled as a single LJ sphere. In the enzyme-enzyme complex, the second enzyme is put behind the active center of the first enzyme, so that it presents only minimal geometrical hindrance for a substrate to access the active site. Our purpose is to see if (and how) the formation of an enzyme-enzyme complex, *i.e.* the complex as such, alters the association rate, and hence the reaction velocity. To calculate the association rates we have used the Northrup-Allison-McCammon algorithm [44, 45] (see Methods).

The results are shown in Fig 3b. Both reaction rates $k_{D}^{ch}$ and $k_{D}^{\left(1\right)}$ decrease as the strength of the interaction between the substrate and the LJ particles of the enzymes, *ϵ*_*LJ*_, increases. This is understandable as the enzymes become ‘less soft’ for the substrate, preventing it from coming close to their active sites, while the van der Waals attraction is too weak to significantly influence the association rate in this case. Since in this case the rate is mainly determined by the substrate diffusion, both rates are comparable (see the inset in Fig 3b), except of high values of *ϵ*_*LJ*_ at which the LJ attraction starts to play a more significant role.

This result suggests *γ*_*ch*_ ≈ *γ*_1_ and hence deceleration of the reaction velocity by channeling in steady-state, according to Fig 1. We note, however, that we have considered uncharged enzymes and that in some cases, *e.g.* when the surface of the second enzyme is charged mainly oppositely to the substrate, an enhancement of $k_{D}^{ch}$ over $k_{D}^{\left(1\right)}$ can be expected. Whether this is sufficient to accelerate the reaction velocity depends on system (*i.e.* on enzyme sizes and charge distributions, substrate concentration *etc.*), and it will be interesting to study such cases explicitly in future work.

## Discussion

We have considered a simple model for substrate channeling, Eq (2). This or similar models have been previously used in the literature in different contexts [10, 15, 29, 30, 46]. The model predicts that in steady state channeling can slow down tandem reactions in a wide range of substrate concentrations, [S]. Such a slow-down is most clearly expressed at high concentrations, at which independently of [S] and of the microscopic rate constants, the channeled reaction is slower than the original non-channeled tandem reaction (Eq (7) and Fig 1). Although this result may seem surprising at first glance, it has a simple explanation. Indeed, in our model we assumed that there is only one metabolite bound to an enzyme-enzyme complex (E_12_ = E_1_E_2_) at a time. This means that E_12_ processes metabolites in series: It first takes a substrate (S) and transforms it into an intermediate (I); next, this intermediate is transfered to the second active site of E_12_, where it is finally converted into the product (P). Only *then* the enzyme-enzyme complex is free to associate with another substrate molecule. In a non-channeled cascade, in contrast, the $\text{S}\overset{{\text{E}}_{1}}{\to }\text{I}$ and $\text{I}\overset{{\text{E}}_{2}}{\to }\text{P}$ reactions can proceed in parallel, provided the concentration of intermediates is sufficiently high. This can in particular be achieved in steady state at high substrate concentrations, as expressed by Eq (7).

Clearly, our model can be criticized for not allowing more than one metabolite in an enzyme-enzyme complex at the same time. It can, however, be easily extended to take this into account, for example, by introducing, in addition to reaction Eq (2), a complementary reaction $\text{S}+{\text{E}}_{\text{12}}\text{I}\overset{a_{ch}}{\underset{d_{ch}}{⇌}}{\text{SE}}_{\text{12}}\text{I}\overset{k_{2}}{\to }{\text{IE}}_{\text{12}}+P,$ where *a*_*ch*_ and *d*_*ch*_ are association/dissociation rate constants and *k*_2_ the turnover number, which we have assumed here to be the same as *k*_1_, for simplicity. This additional reaction describes a process of attaching a substrate to the complex that already contains an intermediate (for instance in the tunnel between its active sites). Thus, the modified model allows two metabolites to be simultaneously present in the complex. In this case we indeed observe an increase in the velocity of the channeled reaction, *viz.* $v_{ch}^{\left(max\right)}=k_{2}k_{ch}\left[E_{12}\right]/\left(k_{2}+k_{ch}\right)$ at high substrate concentrations, but the overall reaction rate is still lower than in the non-channeled system (see Eq (7a)).

One might be tempted to continue this process and allow more and more metabolites in an enzyme-enzyme complex, to see when channeling becomes beneficial for the reaction velocity. It is easy to notice, however, that if the first reaction in a cascade is rate limiting, then, independently of the number of intermediates in the complex, the channeled reaction cannot exceed the product formation velocity set by the first enzyme [15], *i.e.* *k*_1_[E_1_], which is just the production rate of the non-channeled system in steady state at high substrate concentrations (see Eq (7a)).

In the opposite case, when the second reaction in a cascade is rate limiting (*i.e.* *k*_2_ < *k*_1_), it is theoretically possible that the channeled reaction is faster than its non-channeled counterpart (in steady-state). This can happen if channeling increases the local concentration of intermediates at the second enzyme, in comparison to the non-channeled case. Whether this is achievable in practice depends on particular system, substrate concentration, *etc.* However, in this case it is easy to improve the overall rate of the non-channeled system by increasing the concentration of E_2_, to compensate its weak activity. In particular, by taking [E_2_] = *k*_1_[E_1_]/*k*_2_ > [E_1_] we can increase the maximum product formation velocity to $v_{non}^{\left(max\right)}=k_{1}\left[E_{1}\right]$, while the maximum possible rate of the channeled system is $k_{2}\left[E_{12}\right]<v_{non}^{\left(max\right)}$ for [E_12_] = [E_1_]. Obviously, we can also increase the concentration of enzyme-enzyme complexes to [E_12_] = [E_2_], to make a fairer comparison, but this will only lead to the rate as high as $v_{non}^{\left(max\right)}$. Note that in this case the capability of the first enzyme in E_12_ is not fully utilized, unlike it is in the non-channeled system. This illustrates, particularly, that a non-channeled system is more flexible and provides more options for maximizing its product formation velocity.

So far we have discussed only uni-substrate reactions, while many enzymes require more than one substrate or co-factors for the reaction to occur (for instance, many dehydrohynases, oxidases, transferases, etc) [47, 48]. However, it is straightforward to extend our model, Eqs (1) and (2), to take such reactions into account. It can then be shown that, similarly to the case of uni-substrate reactions, channeling can accelerate or decelerate the reaction velocity at low substrate concentrations, depending on the relation between the various rate constants. In saturation, however, when the concentrations of all substrates are high, we obtain the limiting reaction velocities that are similar or identical to Eqs (7a) and (7b), which means that channeling cannot accelerate the reaction velocity in this case (see Supporting Information for bi-substrate reactions).

Thus, our main claim is that channeling may have small or even negative effect on the steady state velocities of cascade reactions. However, enzyme-enzyme complexes do exist *in vivo* [23–27], and it is thus appropriate to ask: What are the benefits of channeling that are possibly exploited by living cells? We shall discuss here two most commonly mentioned assets, *viz.* (i) decreasing the intermediate pool size, which is important *e.g.* to protect cells from toxic intermediates, and (ii) preventing intermediates from side reactions or degradation.

### Decrease of the metabolite pool size

One frequently mentioned advantage of channeling is the decrease of metabolite pool size [32]. This sounds reasonable. Indeed, in the channeled system (with direct channeling) the intermediates are never released into the bulk solution, and thus, one may think, increasing the degree of channeling, *i.e.* the concentration of enzyme-enzyme complexes, [E_12_], should decrease the amount of intermediates in the system. However, Cornish-Bowden and Cárdenas [29] have shown that (in some cases) the concentration of intermediates, [I], is essentially indifferent to the amount of [E_12_]. Mendes *et al*. [30] argued that in more general cases [I] does decrease with increasing [E_12_], and Korzeniewski and Quant [49] demonstrated a simple mechanism to decrease the metabolite pool size by channeling.

To look into this in some details, we consider a system that contains both single enzymes (E_1_ and E_2_) and enzyme-enzyme complexes (E_12_) in varying proportions. It is a simple exercise to calculate the steady-state concentration of intermediates in this system. The result is

$$\begin{array}{c} {\left[\text{I}\right]}_{\text{ss}}=\frac{K_{M}^{\left(2\right)}}{K_{M}^{\left(1\right)}}\frac{k_{1}{\left[E_{1}\right]}_{0}}{k_{2}{\left[E_{2}\right]}_{0}}\frac{\left[S\right]{\left[S\right]}_{\text{threshold}}}{{\left[S\right]}_{\text{threshold}}-\left[S\right]}, \end{array}$$ (11)

where $K_{M}^{\left(i\right)}$ is the Michaelis-Menten constant of enzyme E_*i*_ and [S]_threshold_ is given by Eq (6). The total concentration of single (not complexed with each other) enzymes is [E_*i*_]_0_ = [E_*i*_] − [E_12_], *i* = 1,2.

**Fig 4:**
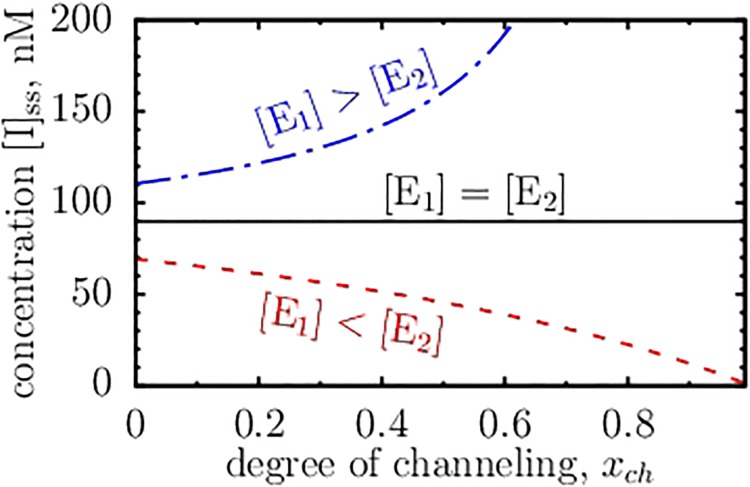
Effect of channeling on the metabolite pool size. The concentration of intermediates in the steady state is shown for systems with varying degree of channeling. The degree of channeling is *x*_*ch*_ = [E_12_]/min([E_1_], [E_2_]), where [E_12_] is the concentration of enzyme-enzyme complexes in the system and [E_*i*_], *i* = 1,2, is the total enzyme concentration (*i.e.* free, not complexed E_*i*_ plus E_12_). The concentration [E_1_] = 50 nM and the rate constant *k*_1_ = 1.5 s^−1^ are the same in all three systems. The concentration of [E_2_] and the rate constant *k*_2_ are adjusted to satisfy *k*_1_[E_1_] = *k*_2_[E_2_], in which case the enzymes capabilities are fully utilized. We chose *k*_2_ = 1.5 s^−1^ = *k*_1_ and [E_2_] = 50 nM = [E_1_] (solid line); *k*_2_ = 1 s^−1^ < *k*_1_ and [E_2_] = 75 nM >[E_1_] (red dash line); and *k*_2_ = 2 s^−1^ > *k*_1_ and [E_2_] = 37.5 nM < [E_1_] (dot-dash blue line). The degree of channeling *γ*_1_ = *γ*_2_ = *γ*_*ch*_ = 1 (see Eq (10)). The substrate concentration ([S] = 90 nM) and the remaining rate constants are the same as in Fig 1.

It is convenient to define the degree of channeling *x*_*ch*_ = [E_12_]/min([E_1_], [E_2_]). Now, it directly follows from Eq (11) that for [E_1_] = [E_2_] the steady-state concentration of intermediates is independent of *x*_*ch*_. This corresponds to the Cornish-Bowden–Cárdenas case and is shown by the solid line in Fig 4. The dash line in this figure shows the case when channeling reduces the amount of intermediates in the system. But, inverse is also possible: For [E_1_] > [E_2_], [I] increases as the degree of channeling increases (dash-dot line in Fig 4). Such a scenario has already been pointed out by Cornish-Bowden [50].

This result can be easily understood. Assume *k*_1_ = *k*_2_/2 and imagine the extreme case of just two E_1_ enzymes and one E_2_ enzyme, so that *k*_1_[E_1_] = *k*_2_[E_2_], as in Fig 4 (this relation gives the enzyme concentrations that optimize an enzymatic cascade in saturation). When E_1_ and E_2_ form a complex, some metabolites are channeled by E_12_, but the remaining E_1_ produces the intermediates that cannot be converted into the product; thus, [I] increases to infinity as we approach the steady state. In a less extreme case, we observe a finite increase of [I] as E_1_ and E_2_ dimerize into a complex. This can also be easily seen from Eq (11), which gives *d*[I]_ss_/*dx*_*ch*_ ∼ [E_2_] − [E_1_]. Interestingly, the sign of *d*[I]_ss_/*dx*_*ch*_ does not depend on the reaction constants, and thus channeling *always* increases the metabolite pool size in steady state if the first enzyme is abundant with respect to the second. Since we have not made any specific assumption on the enzyme environment, this conclusion should hold *in vitro* as well as *in vivo*.

### Protecting intermediates from degradation or competing reactions

In living cells or in catalytic reactors, it may happen that intermediates degrade, decompose or are consumed by competing side reactions. To model this situation, it is usual to supplement the evolution equation for the intermediates by a term *k*_deg_[I], where *k*_deg_ is the effective degradation rate constant. Then the product formation velocity in the steady state is $v_{non}=v_{non}^{\left(0\right)}-k_{\text{deg}}{\left[\text{I}\right]}_{\text{ss}}$, where $v_{non}^{\left(0\right)}$ corresponds to *k*_deg_ = 0 and is given by Eq (4), and [I]_ss_ is the steady-state concentration of intermediates (note that [I]_ss_ is not given by Eq (11); it depends on *k*_deg_ and must be found from a quadratic equation, which we solved numerically, see Methods).

**Fig 5:**
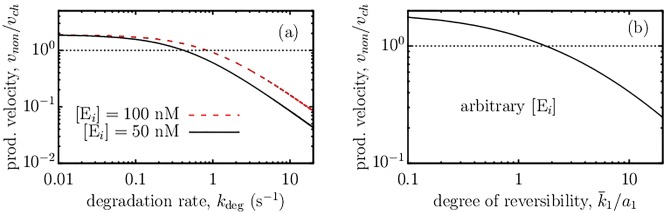
Effect of channeling on reaction cascades with competing reactions. (a) Product formation velocity of the nonchanneled system, *v*_*non*_, decreases as the degradation or consumption rate of intermediates, *k*_deg_, increases. (b) Reaction velocity of the nonchanneled system falls off for increasing ‘degree of reversibility’ of the first reaction in the cascade, ${\bar{k}}_{1}/a_{1}$. In this case the reaction is $\text{S}+{\text{E}}_{\text{1}}\overset{a_{1}}{\underset{d_{1}}{⇌}}{\text{SE}}_{\text{1}}\overset{k_{1}}{\underset{\underset{{\bar{k}}_{1}}{↽}}{⇀}}{\text{E}}_{\text{1}}+\text{I}$ in lieu of Eq (1a). In both plots *v*_*non*_ is compared to the velocity of the channeled system, *v*_*ch*_. The concentrations of enzymes and enzyme-enzyme complexes are equal in both systems on both panels, *i.e.* [E_*i*_] = [E_1_] = [E_2_] = [E_12_]; in panel (b) the ratio *v*_*non*_/*v*_*ch*_ is independent of [E_*i*_]. Substrate concentration [S] = 90 nM, the degree of diffusion control is *γ*_1_ = *γ*_*ch*_ = 1 (see Eq (10)), and the rate constants are the same as in Fig 1. Thin dotted line denotes *v*_*ch*_ = *v*_*non*_.

Fig 5a compares the product formation velocity in the non-channeled and channeled systems (we assume that the latter is unaffected by *k*_deg_). We have chosen the rate constants such that the channeled system is slower when *k*_deg_ = 0 (see Fig 1). As one may expect, the product formation velocity decreases as *k*_deg_ increases, however, this decrease becomes significant only when *k*_deg_ ≳ 1 s^−1^ (for the parameters of the plot). The velocity of the non-channeled system can be improved by increasing the concentration of enzymes, but this does not rescue the system from low production velocities at high *k*_deg_.

A particularly interesting example of competing reactions is a reversible reaction I ⇌ S catalyzed by the same enzyme (E_1_); such enzymes are numerous and include many important isomerases, aldolases, dehydrogenases, transferases and others. The peculiarity of this system is that the reverse reaction, although competing, transforms the intermediates back into the substrates, which can then be reused; however, this process effectively reduces the amount of enzyme E_1_ available for the direct reaction. To study this case, we consider, instead of reaction Eq (1a), a one-complex reversible reaction $\text{S}+{\text{E}}_{\text{1}}\overset{a_{1}}{\underset{d_{1}}{⇌}}{\text{SE}}_{\text{1}}\overset{k_{1}}{\underset{\underset{{\bar{k}}_{1}}{↽}}{⇀}}{\text{E}}_{\text{1}}+\text{I}$, and leave the channeled reaction unaltered (in a more general case of two-complex reversible reactions the channeled reaction will also be affected by reversibility). Fig 5b shows that the non-channeled reversible reaction provides higher rates for low degrees of reversibility, ${\bar{k}}_{1}/a_{1}\;≲\;1$, while channeling becomes beneficial as ${\bar{k}}_{1}$ increases. Unlike it is in the case of degradation of intermediates (Fig 5a), the ratio of the velocities of the channeled and non-channeled reactions is independent of the enzyme concentration, provided of course [E_1_] = [E_2_] = [E_12_].

## Conclusions

Using a simple reaction-kinetics model, supported by Brownian dynamics simulations, we have shown that metabolite channeling does not necessarily accelerate cascade reactions and can even decelerate them (Fig 1). In addition, we demonstrated that the metabolite pool size can increase as the degree of channeling increases (Fig 4). We corroborated however that channeling provides a convenient way to increase the velocity of a cascade reaction if intermediates degrade or participate in competing reactions (Fig 5). This suggests therefore that the main advantage of channeling is unlikely the reaction acceleration or decrease of the metabolite pool size, but it rather lies in protecting intermediates from degradation or competing reactions.

Our study is by no means conclusive and complete, however. Indeed, we considered a simple homogeneous mean-field model and ignored any chemical details of enzymes and metabolites. This may be important in some situations and deserves separate studies. For instance, we performed Brownian dynamics simulations of the diffusion-controlled rates for a model system (Fig 3), and it is desirable to perform a similar study for real enzymes and enzyme-enzyme complexes. Additionally, it will be interesting to investigate the effect of diffusion slow-down [51–53] and anomalous diffusion [54–58] on channeling, which is highly important for living systems [59]. It will be particularly interesting to see how channeling influences the response of a system to external stimuli [60–62], where channeling can potentially be advantageous.

Experimental studies have mainly focused on artificial enzyme complexes and channeling by proximity [20, 21, 32, 63], while experiments on direct channeling are currently scarce. Such experiments would ideally provide a careful examination of microscopic association/dissociation rates and turnover numbers of complexed and ‘free’ enzymes, and should be performed under steady-state, rather than typically used laboratory initial conditions. While the latter is relatively easy to achieve, *e.g.* by constantly supplying substrates to the system or using the steady-state concentration of intermediates from the onset of a measurement, the former is challenging and demands significant advances in the experimental techniques and theory.

Thus, the question posed in the title of this article requires further and more complex theoretical and, in particular, experimental investigations, and we hope that our work will stimulate more active research and discussions in this direction.

## Methods

### Homogeneous well-mixed systems

We consider a mixed system containing both enzymes E_1_ and E_2_ and the enzyme-enzyme complexes E_12_ = E_1_E_2_. To obtain a fully channeled or a pure non-channeled system, we set the concentrations [E_1_] = [E_2_] = 0 or [E_12_] = 0, respectively, at all times.

Applying the mass action law to reactions Eqs (1) and (2), we obtain a system of ordinary differential equations, which is straightforward but lengthy, and is not shown here in full to save space (see Supporting Information for the whole set of equations). The first equation of this system is

$$\begin{array}{c} \frac{d[S]}{dt}=-a_{1}\left[E_{1}\right]\left[S\right]+d_{1}\left[SE_{1}\right]-a_{ch}\left[E_{12}\right]\left[S\right]+d_{ch}\left[SE_{12}\right]+v_{S}, \end{array}$$ (12)

where *v*_*S*_ is the substrate supply velocity. In a batch system *v*_*S*_ = 0, while in a fed-bach system it is non-zero. In a system where the substrate concentration is kept constant, *d*[S]/*dt* = 0, and *v*_*S*_ = *v*_*S*_(*t*) ≠ 0 is in general not constant, but attains a constant value in the steady state as *t* → ∞.

In addition to the differential equations discussed above, there are also conservation laws for the enzyme and metabolite concentrations. For instance, [E_1_]_tot_ = [E_1_](*t*) + [SE_1_](*t*), where [E_1_]_tot_ is the total concentration of enzyme E_1_ (not dimerized with E_2_); it is equal to the free (*i.e.* not complexed) concentration of E_1_ at time *t* = 0, *i.e.* [E_1_]_tot_ = [E_1_](*t* = 0), and similarly for other species. (The total enzyme concentrations are used on all plots in the main text, but we remove ‘tot’ to avoid clumsy notations.) This reduces the number of independent variables from 11 to 7 (in the mixed system). Nevertheless, it is difficult to solve this system of equations analytically and we used OCTAVE with Hindmarsh’s solver LSODE [64] to obtain the time-dependent profiles of various species in the system numerically.

In steady state, all concentrations approach constant values and the differential equations reduce to a system of algebraic equations, which we have solved analytically. (This reasoning applies to fed-batch and constant substrate concentration conditions, in which, however, the product concentration increases with time. Additionally, it is possible that [I] → ∞ or [S] → ∞ as *t* → ∞. This occurs when the substrate supply velocity is too high for the first enzyme to deal with the available substrates, and when the first enzyme is more efficient than the second enzyme so that the second enzyme is unable to deal with the increasing amount of intermediates. The condition when this happens can be readily obtained and is given by Eq (6).) The resulting equations are presented and discussed in the main text.

The evolution equation for the intermediates reads

$$\begin{array}{c} \frac{d[\text{I}]}{dt}=k_{1}\left[SE_{1}\right]-a_{2}\left[E_{2}\right]\left[\text{I}\right]+d_{2}\left[\text{I}E_{2}\right]-k_{\text{deg}}\left[\text{I}\right], \end{array}$$ (13)

where *k*_deg_ is the rate due to degradation or competing side reactions (*i.e.* in addition to reactions Eqs (1) and (2) we have $\text{I}\overset{k_{\text{deg}}}{\to }\emptyset$). In steady state, the product formation velocity of the non-channeled system is $v_{non}=v_{non}^{\left(0\right)}-k_{\text{deg}}{\left[\text{I}\right]}_{\text{ss}}$, where $v_{non}^{\left(0\right)}$ denotes the production velocity when *k*_deg_ = 0 and is given by Eq (4). [I]_ss_ is the steady-state concentration of intermediates and satisfies the following equation

$$\begin{array}{c} v_{non}^{\left(0\right)}-\frac{k_{2}{\left[\text{I}\right]}_{\text{ss}}\left[E_{2}\right]}{K_{M}^{\left(2\right)}+{\left[\text{I}\right]}_{\text{ss}}}-k_{\text{deg}}{\left[\text{I}\right]}_{\text{ss}}=0. \end{array}$$ (14)

In the case when the *first* reaction in the cascade is one-complex reversible, the product formation velocity in the steady state is

$$\begin{array}{c} v_{non}=\frac{k_{2}\left[E_{2}\right]{\left[\text{I}\right]}_{\text{ss}}}{{\left[\text{I}\right]}_{\text{ss}}+K_{M}^{\left(2\right)}}, \end{array}$$ (15)

where the steady-state concentration of intermediates satisfies

$$\begin{array}{c} v_{non}=\frac{\left[E_{1}\right]}{1+\left[S\right]/K_{M}^{\left(1\right)}+{\left[\text{I}\right]}_{\text{ss}}/{\bar{K}}_{M}^{\left(1\right)}}\left\{\frac{k_{1}\left[S\right]}{K_{M}^{\left(1\right)}}-\frac{d_{1}{\left[\text{I}\right]}_{\text{ss}}}{{\bar{K}}_{M}^{\left(1\right)}}\right\}, \end{array}$$ (16)

where ${\bar{K}}_{M}^{\left(1\right)}=\left(k_{1}+d_{1}\right)/{\bar{k}}_{1}$ and ${\bar{k}}_{1}$ is the rate of the reverse reaction ${\text{E}}_{1}+\text{I}\overset{{\bar{k}}_{1}}{\underset{k_{1}}{⇌}}{\text{SE}}_{\text{1}}$. We solved Eqs (14) and (16) numerically using the GNU Scientific Library [65].

### Diffusion-controlled rates

We have used the ready-to-use software package browndye written by Gary Huber [66] to calculate the association rates between a substrate molecule and an enzyme or a complex of two enzymes.

Enzymes were created by placing small Lennard-Jones spheres (of radius ≈0.37 nm) on the nodes of a triangularized sphere of radius 1.5 nm. One of the small spheres was declared an active site. The triangularization was done using the GTS library [67]. An enzyme-enzyme complex was created by merging two identical enzymes such that their active sites were opposite to each other on the line connecting the centers of the two enzymes (the active site of the second enzyme in the complex did not participate in simulations, however). The distance between the enzyme centers was 1.4 nm. The substrate was a Lennard-Jones sphere of radius 0.4 nm. The enzymes and substrates were not charged.

The Northrup-Allison-McCammon algorithm was used to calculate the diffusion-limited reaction rates [44, 45]. In this algorithm the substrate and the enzyme are initially separated by some distance called *b*-radius and the reaction rate is calculated by estimating the probability of a substrate molecule to approach the active site of the enzyme to within a user specified reaction distance (taken 0.82 nm in our simulations). The *b*-radius was 5 nm and 10^5^ independent runs (trajectories) were analyzed. In a few cases, up to 10^7^ runs were used, leading to no significant differences.

## Supporting information

S1 Appendix Evolution equations for enzyme-catalyzed cascade reactions. This appendix includes the full set of ordinary differential equations for the evolution of concentrations of various species in the well-mixed homogeneous system of enzyme-catalyzed uni-substrate tandem reaction and its channeled counterpart. In addition it contains the steady-state equations for enzyme-catalyzed bi-substrate tandem reactions and their channeled versions; these include sequential ordered and random reactions as well as ping-pong (double-displacement) reactions.
(PDF)
