# Supplementary material for: Does metabolite channeling accelerate enzyme-catalyzed cascade reactions?
Source: PLoS One. 2017 Feb 24;12(2):e0172673. doi: 10.1371/journal.pone.0172673 (PMC5325314; doi:10.1371/journal.pone.0172673)
Supplement: S1 Appendix — This appendix includes the full set of ordinary differential equations for the evolution of concentrations of various species in the well-mixed homogeneous system of enzyme-catalyzed uni-substrate tandem reaction and its channeled counterpart. In addition it contains the steady-state equations for enzyme-catalyzed bi-substrate tandem reactions and their channeled versions; these include sequential ordered and random reactions as well as ping-pong (double-displacement) reactions. (PDF) [file pone.0172673.s001.pdf]

Does metabolite channeling accelerate enzyme-catalyzed cascade reactions? Supporting Information.

Liubov Poshyvailo, Eric von Lieres, and Svyatoslav Kondrat

S1. EVOLUTION EQUATIONS FOR UNI-SUBSTRATE REACTIONS

Applying the law of mass action to Eqs. (1) and (2) of the main text, we obtain a system of ordinary differential equations, which defines the evolution of concentrations of various species in the system. The equation for the substrate concentration reads

$$\frac{d[\mathrm{S}]}{dt} = -a_1[\mathrm{E}_1][\mathrm{S}] + d_1[\mathrm{SE}_1] - a_{ch}[\mathrm{E}_{12}][\mathrm{S}] + d_{ch}[\mathrm{SE}_{12}] + v_S, \tag{1}$$

which is Eq. (12) of the main text. The concentrations of enzymes, intermediates and Michaelis-Menten complexes of the non-channeled subsystem satisfy

$$\frac{d[\mathrm{E}_1]}{dt} = d_1[\mathrm{E}_1\mathrm{S}] - a_1[\mathrm{E}_1][\mathrm{S}] + k_1[\mathrm{SE}_1] \tag{2a}$$

$$\frac{d[\mathrm{SE}_1]}{dt} = a_1[\mathrm{E}_1][\mathrm{S}] - d_1[\mathrm{SE}_1] - k_1[\mathrm{SE}_1] \tag{2b}$$

$$\frac{d[\mathrm{I}]}{dt} = k_1[\mathrm{SE}_1] - a_2[\mathrm{E}_2][\mathrm{I}] + d_2[\mathrm{IE}_2] - k_{\mathrm{deg}}[\mathrm{I}] \tag{2c}$$

$$\frac{d[\mathrm{E}_2]}{dt} = d_2[\mathrm{IE}_2] - a_2[\mathrm{E}_2][\mathrm{I}] + k_2[\mathrm{IE}_2] \tag{2d}$$

$$\frac{d[\mathrm{IE}_2]}{dt} = a_2[\mathrm{E}_2][\mathrm{I}] - d_2[\mathrm{IE}_2] - k_2[\mathrm{IE}_2]. \tag{2e}$$

The evolution equations for the channeled subsystem are

$$\frac{d[\mathrm{E}_{12}]}{dt} = d_{ch}[\mathrm{SE}_{12}] - a_{ch}[\mathrm{S}][\mathrm{E}_{12}] + k_2^{ch}[\mathrm{E}_{12}\mathrm{I}] \tag{3a}$$

$$\frac{d[\mathrm{SE}_{12}]}{dt} = a_{ch}[\mathrm{S}][\mathrm{E}_{12}] - d_{ch}[\mathrm{SE}_{12}] - k_1^{ch}[\mathrm{SE}_{12}] \tag{3b}$$

$$\frac{d[\mathrm{IE}_{12}]}{dt} = k_1^{ch}[\mathrm{SE}_{12}] - k_{ch}[\mathrm{IE}_{12}] \tag{3c}$$

$$\frac{d[\mathrm{E_{12}I}]}{dt} = k_{ch}[\mathrm{IE_{12}}] - k_2^{ch}[\mathrm{E_{12}I}]. \tag{3d}$$

Finally, the product formation velocity is

$$v_P = \frac{d[\mathrm{P}]}{dt} = k_2[\mathrm{IE_2}] + k_2^{ch}[\mathrm{E_{12}I}]. \tag{4}$$

These evolution equations must be supplemented by the mass conservation. For the non-channeled subsystem we have

$$[\mathrm{E_1}]_{\mathrm{tot}} = [\mathrm{E_1}] + [\mathrm{SE_1}] \tag{5a}$$

$$[\mathrm{E_2}]_{\mathrm{tot}} = [\mathrm{E_2}] + [\mathrm{IE_2}], \tag{5b}$$

where $[\mathrm{E}_i]_{\mathrm{tot}}$ is the total (supplied) concentration of E_i $(i = 1, 2)$, and

$$[\mathrm{E_{12}}]_{\mathrm{tot}} = [\mathrm{E_{12}}] + [\mathrm{SE_{12}}] + [\mathrm{IE_{12}}] + [\mathrm{E_{12}I}], \tag{6}$$

for the channeled subsystems. We note that the total enzyme concentrations ($[\mathrm{E}_i]_{\mathrm{tot}}$ and $[\mathrm{E_{12}}]_{\mathrm{tot}}$) are shown on all plots in the main text, where we skip 'tot' to avoid clumsy notations. For the substrate we have

$$[\mathrm{S}]_0 = [\mathrm{S}] + [\mathrm{SE_1}] + [\mathrm{IE_2}] + [\mathrm{I}] + [\mathrm{SE_{12}}] + [\mathrm{IE_{12}}] + [\mathrm{E_{12}I}] + [\mathrm{P}] - v_S t, \tag{7}$$

where $[\mathrm{S}]_0$ is the initial substrate concentration. Thus, we have in total a system of seven independent equations.

S2. BI-SUBSTRATE REACTIONS

We consider here the situation when the first reaction in a cascade is bi-substrate. For simplicity we limit our considerations to irreversible transformations, but include dissociations as before. We shall also assume that for sequential (single displacement) reactions, the rate of transformation/dissociation of the second product of the first enzyme (if any) is fast, as compared to k_1 (see below), so that the second product can be ignored.

13 A. Sequential ordered reactions

We consider the following cascade

$$\mathrm{S_1 + E_1 \underset{d_{11}}{\overset{a_{11}}{\rightleftharpoons}} S_1E_1}, \tag{8a}$$

$$\mathrm{S_2 + S_1E_1 \underset{d_{12}}{\overset{a_{12}}{\rightleftharpoons}} S_2S_1E_1 \xrightarrow{k_1} E_1 + I}, \tag{8b}$$

$$\mathrm{I + E_2 \underset{d_2}{\overset{a_2}{\rightleftharpoons}} IE_2 \xrightarrow{k_2} E_2 + P}, \tag{8c}$$

with the mass conservation for enzyme $\mathrm{E_1}$

$$[\mathrm{E_1}]_{\mathrm{tot}} = [\mathrm{E_1}] + [\mathrm{S_1E_1}] + [\mathrm{S_2S_1E_1}]. \tag{8d}$$

The corresponding channeled reaction reads

$$\mathrm{S_1 + E_{12} \underset{d_{11}^{ch}}{\overset{a_{11}^{ch}}{\rightleftharpoons}} S_1E_{12}}, \tag{9a}$$

$$\mathrm{S_2 + S_1E_{12} \underset{d_{12}^{ch}}{\overset{a_{12}^{ch}}{\rightleftharpoons}} S_2S_1E_{12} \xrightarrow{k_1} IE_{12} \xrightarrow{k_{ch}} E_{12}I \xrightarrow{k_2} E_{12} + P}. \tag{9b}$$

The evolution equation can be readily written down and solved in the steady state approximation, assuming that the steady state concentration of intermediates $[\mathrm{I}]_{\mathrm{ss}} < \infty$. The result is (in the double reciprocal form and removing tot in $[\mathrm{E_1}]_{\mathrm{tot}}$ and $[\mathrm{E_{12}}]_{\mathrm{tot}}$ for brevity)

$$\frac{1}{v_{non}} = \frac{1}{k_1[\mathrm{E_1}]}\left\{1 + \frac{K_M^{(12)}}{[\mathrm{S_2}]} + \frac{k_1}{a_{11}}\frac{1}{[\mathrm{S_1}]} + \frac{K_M^{(12)}K_S^{(11)}}{[\mathrm{S_1}][\mathrm{S_2}]}\right\}, \tag{10}$$

where $K_S^{(11)} = d_{11}/a_{11}$ and $K_M^{(12)} = (k_1 + d_{12})/a_{12}$; the velocity of the channeled reaction

$$\frac{1}{v_{ch}} = \frac{1}{k_1[\mathrm{E_{12}}]}\left\{\frac{k_1}{k_{\mathrm{tot}}^{ch}} + \frac{K_M^{ch}}{[\mathrm{S_2}]} + \frac{k_1}{a_{11}^{ch}}\frac{1}{[\mathrm{S_1}]} + \frac{K_M^{ch}K_S^{ch}}{[\mathrm{S_1}][\mathrm{S_2}]}\right\}, \tag{11}$$

14 where $1/k_{\mathrm{tot}}^{ch} = 1/k_1 + 1/k_2 + 1/k_{ch}$, $K_M^{ch} = (k_1 + d_{12}^{ch})/a_{12}^{ch}$, and $K_S^{ch} = d_{11}^{ch}/a_{11}^{ch}$.

Similarly as in the case of uni-substrate reactions, channeling can accelerate or decelerate the reaction velocity at low substrate concentrations, depending on the relation between $K_M^{(12)}$, $K_S^{(11)}$, K_M^{ch} and K_S^{ch}. At high concentrations of $[\mathrm{S_1}] \gg \max(k_1/a_{11}, K_M^{(12)}K_S^{(11)}/[\mathrm{S_2}], k_1/a_{11}^{ch}, K_M^{ch}K_S^{ch}/[\mathrm{S_2}])$, Eqs. (10) and (11) reduce to the uni-substrate case (Eqs. (3) and (4) of the main text). If $[\mathrm{S_2}] \gg K_M^{(12)}$ and $[\mathrm{S_2}] \gg K_M^{ch}$ but $[\mathrm{S_1}]$ is finite, then we obtain that channeling accelerates the reaction velocity if

$$\gamma_{11}^{ch}/\gamma_{11} < 1 - \frac{k_a^{(11)}[\mathrm{S_1}]}{\gamma_{11}}\left(\frac{1}{k_2} + \frac{1}{k_{ch}}\right), \tag{12}$$

where γ_{11} and γ_{11}^{ch} are the degrees of the diffusion control of the first reaction in the cascade and its channeled counterpart, respectively, and $k_a^{(11)}$ is the corresponding microscopic association rate constant, taken the same for the channeled and non-channeled reactions (see Eqs. (10) of the main text).

Finally, when both $[S_1]$ and $[S_2]$ are high, we recover the maximum reaction velocities given by Eqs. (7a) and (7b) of the main text.

B. Sequential random reactions

We consider the following cascade

$$S_1 + E_1 \underset{d_{11}}{\overset{a_{11}}{\rightleftharpoons}} S_1E_1, \tag{13a}$$

$$S_2 + E_1 \underset{d_{12}}{\overset{a_{12}}{\rightleftharpoons}} S_2E_1, \tag{13b}$$

$$S_1 + S_2E_1 \underset{\tilde{d}_{11}}{\overset{\tilde{a}_{11}}{\rightleftharpoons}} S_1S_2E_1, \tag{13c}$$

$$S_2 + S_1E_1 \underset{\tilde{d}_{12}}{\overset{\tilde{a}_{12}}{\rightleftharpoons}} S_1S_2E_1, \tag{13d}$$

$$S_1S_2E_1 \xrightarrow{k_1} E_1 + I, \tag{13e}$$

$$I + E_2 \underset{d_2}{\overset{a_2}{\rightleftharpoons}} IE_2 \xrightarrow{k_2} E_2 + P, \tag{13f}$$

with the mass conservation for enzyme E_1

$$[E_1]_{tot} = [E_1] + [S_1E_1] + [S_2E_1] + [S_2S_1E_1]. \tag{13g}$$

The corresponding channeled reaction reads

$$S_1 + E_{12} \underset{d_{11}^{ch}}{\overset{a_{11}^{ch}}{\rightleftharpoons}} S_1E_{12}, \tag{14a}$$

$$S_2 + E_{12} \underset{d_{12}^{ch}}{\overset{a_{12}^{ch}}{\rightleftharpoons}} S_2E_{12}, \tag{14b}$$

$$S_1 + S_2E_{12} \underset{\tilde{d}_{11}^{ch}}{\overset{\tilde{a}_{11}^{ch}}{\rightleftharpoons}} S_1S_2E_{12}, \tag{14c}$$

$$S_2 + S_1E_{12} \underset{\tilde{d}_{12}^{ch}}{\overset{\tilde{a}_{12}^{ch}}{\rightleftharpoons}} S_1S_2E_{12}, \tag{14d}$$

$$S_1S_2E_{12} \xrightarrow{k_1} IE_{12} \xrightarrow{k_{ch}} E_{12}I \xrightarrow{k_2} E_{12} + P. \tag{14e}$$

Random reactions are slightly more complex and the resulting equations are cumbersome and little appealing. To simplify them we assume rapid equilibrium for the first two reactions, *i.e.* for reactions (13a) and (13b) and their channeled counterparts (14a) and (14b). In this case we readily obtain

$$\frac{1}{v_{non}} = \frac{1}{k_1[\mathrm{E}_1]}\left\{1 + \frac{K_M^{(1)}}{K_{11}[\mathrm{S}_2]} + \frac{K_M^{(1)}}{K_{12}[\mathrm{S}_1]} + \frac{K_M^{(1)}}{[\mathrm{S}_1][\mathrm{S}_2]}\right\}, \tag{15}$$

where

$$K_M^{(1)} = \frac{\tilde{d}_{11} + \tilde{d}_{12} + k_1}{\tilde{a}_{11}/K_{12} + \tilde{a}_{12}/K_{11}} \tag{16}$$

and $(i = 1, 2)$

$$K_{1i} = \frac{d_{1i}}{a_{1i}}. \tag{17}$$

For the channeled reaction we get

$$\frac{1}{v_{ch}} = \frac{1}{k_1[\mathrm{E}_{12}]}\left\{\frac{k_1}{k_{\mathrm{tot}}^{ch}} + \frac{K_M^{ch}}{K_{11}^{ch}[\mathrm{S}_2]} + \frac{K_M^{ch}}{K_{12}^{ch}[\mathrm{S}_1]} + \frac{K_M^{ch}}{[\mathrm{S}_1][\mathrm{S}_2]}\right\}, \tag{18}$$

where $1/k_{\mathrm{tot}}^{ch} = 1/k_1 + 1/k_2 + 1/k_{ch}$ as before,

$$K_M^{ch} = \frac{\tilde{d}_{11}^{ch} + \tilde{d}_{12}^{ch} + k_1}{\tilde{a}_{11}^{ch}/K_{12}^{ch} + \tilde{a}_{12}^{ch}/K_{11}^{ch}} \tag{19}$$

and $(i = 1, 2)$

$$K_{1i}^{ch} = \frac{d_{1i}^{ch}}{a_{1i}^{ch}}. \tag{20}$$

It follows from these equations that for finite substrate concentrations, or when only one substrate is in saturation, channeling can accelerate or decelerate the reaction velocity, depending on the relations between the various rate constants. However, if the concentration of both substrates is high, *viz.* $[\mathrm{S}_i] \gg K_M^{(1)}$ and $[\mathrm{S}_i] \gg K_M^{ch}$, we recover the case of uni-substrate reactions given by Eqs. (7a) and (7b) of the main text.

C. Ping-pong reactions

In ping-pong or double-displacement reactions, the first substrate 'activates' the enzyme (*e.g.* by leaving a functional group at the active center of the enzyme), and the second

substrate is transformed into an intermediate, which can be channeled. The tandem reaction has the form

$$S_1 + E_1 \underset{d_{11}}{\overset{a_{11}}{\rightleftharpoons}} S_1E_1 \xrightarrow{k_{11}} E_1^* + I^*, \tag{21a}$$

$$S_2 + E_1^* \underset{d_{12}}{\overset{a_{12}}{\rightleftharpoons}} S_2E_1^* \xrightarrow{k_{12}} E_1 + I, \tag{21b}$$

$$I + E_2 \underset{d_2}{\overset{a_2}{\rightleftharpoons}} IE_2 \xrightarrow{k_2} E_2 + P, \tag{21c}$$

with the mass conservation for enzyme E_1

$$[E_1]_{tot} = [E_1] + [S_1E_1] + [E_1^*] + [S_2E_1^*]. \tag{21d}$$

The channeled reaction is

$$S_1 + E_{12} \underset{d_{11}^{ch}}{\overset{a_{11}^{ch}}{\rightleftharpoons}} S_1E_{12} \xrightarrow{k_{11}} E_{12}^* + I_*, \tag{22a}$$

$$S_2 + E_{12}^* \underset{d_{12}^{ch}}{\overset{a_{12}^{ch}}{\rightleftharpoons}} S_2E_{12}^* \xrightarrow{k_{12}} IE_{12}^* \xrightarrow{k_{ch}} E_{12}^*I \xrightarrow{k_2} E_{12} + P, \tag{22b}$$

where the mass conservation is

$$[E_{12}]_{tot} = [E_{12}] + [S_1E_{12}] + [E_{12}^*] + [S_2E_{12}^*] + [IE_{12}^*] + [E_{12}^*I]. \tag{22c}$$

The steady-state reaction velocities can be readily calculated and the result is

$$\frac{1}{v_{non}} = \frac{1}{[E_1]}\left\{\frac{1}{k_{11}} + \frac{1}{k_{12}} + \frac{K_M^{(11)}}{k_{11}[S_1]} + \frac{K_M^{(12)}}{k_{12}[S_2]}\right\}, \tag{23}$$

where $(i = 1, 2)$

$$K_M^{(1i)} = \frac{d_{1i} + k_{1i}}{a_{1i}}. \tag{24}$$

For the channeled reaction we get

$$\frac{1}{v_{non}} = \frac{1}{[E_1]}\left\{\frac{1}{k_{tot}^{ch}} + \frac{K_{M,ch}^{(11)}}{k_{11}[S_1]} + \frac{K_{M,ch}^{(12)}}{k_{12}[S_2]}\right\}, \tag{25}$$

where $1/k_{tot}^{ch} = 1/k_{11} + 1/k_{12} + 1/k_2 + 1/k_{ch}$ and $(i = 1, 2)$

$$K_{M,ch}^{(1i)} = \frac{d_{1i}^{ch} + k_{1i}}{a_{1i}^{ch}}. \tag{26}$$

Similarly as in all other cases, at low substrate concentrations, channeling can accelerate or decelerate the reaction velocity, depending on the relation between $K_M^{(1i)}$ and $K_{M,ch}^{(1i)}$. At high concentrations of both substrates we have

$$v_{non}^{(max)} = [\mathrm{E}_1]\,(1/k_{11} + 1/k_{12})^{-1} \tag{27}$$

and

$$v_{ch}^{(max)} = k_{\mathrm{tot}}^{ch}[\mathrm{E}_1]. \tag{28}$$

Clearly, $v_{ch}^{(max)} \leq v_{non}^{(max)}$, as for the uni-substrate reactions. Note that for ping-pong reactions we have the inverse sum, $1/k_{11} + 1/k_{12}$, instead of the single reaction constant k_1 (compare Eqs. (27) and (28) with Eqs. (7a) and (7b) of the main text).
